# In search for the role of thermospermine synthase gene in poplar vascular development

**DOI:** 10.1186/1753-6561-5-S7-P72

**Published:** 2011-09-13

**Authors:** Ana Milhinhos, Andreia Matos, Francisco Vera-Sirera, Miguel Blazquez, Célia Miguel

**Affiliations:** 1 Instituto de Tecnologia Química e Biológica-Univ. Nova de Lisboa; Instituto de Biologia Experimental e Tecnológica (ITQB-UNL;IBET) Av. República, EAN, 2780-157 Oeiras, Portugal; 2 Instituto de Biología Molecular y Celular de Plantas (CSIC-UPV) 46022 Valencia, Spain

## Background

Plant polyamines are preferentially detected in actively growing tissues and have been implicated in growth and developmental processes such as embryogenesis, floral developmental, fruit ripening, senescence and stress responses [1]. Recently it has been established a link between polyamines and vascular development as it was found that, in *Arabidopsis,* the loss-of-function mutants of *ACAULIS5* (*ACL5*) gene, encoding thermospermine synthase, exhibit a severe dwarf phenotype, suggesting that thermospermine acts as a regulator of stem elongation [2,3]. However, in trees, no studies have yet been reported. Due to the relevance of vascular development in wood formation we are investigating the role of thermospermine in vascular tissues of poplar.

## Materials and methods

A search for *ACL5*-like sequences in *Populus trichocarpa* genome allowed us to identify three putative *ACL5* orthologous genes. Based on the degree of sequence similarity, we have selected one of them, *PtACL5*, to generate transgenic plants bearing the constructs for overexpression and silencing of this gene in poplar.

## Results

High expression levels of *PtACL5* in overexpression transgenic lines have been found to be correlated to higher thermospermine content in leaves and young stems, but not to a higher level of other polyamines, suggesting that *PtACL5* encodes a thermospermine synthase in poplar, and it is most probably an ortholog of *ACL5* in poplar. Interestingly, these plants display altered and arrested shoot development in the early stages following p35S::Pt*ACL5* transformation, as well as severe dwarfism. Anatomical changes associated to the lack of elongation include arrested development of the root system and no elongation of the stem from the first internode onwards. Because *Arabidopsisacl5* loss-of-function mutants show accelerated vessel cell death and *ACL5* expression is confined to xylem vessel elements [4], we further looked for alterations in the vascular pattern of the poplar stem, and observed the development of a wider stem in dwarf plants, composed of primary vascular tissues only, lower number of metaxylem cells and with no secondary growth.

## Conclusions

Overall, our results suggest that thermospermine has a regulatory role in xylem differentiation/maturation in poplar. Although a feedback control of thermospermine synthesis seems to be present in *Arabidopsis*, in our transgenic poplar the high levels resulting from overexpression of thermospermine synthase gene seem to overcome any turn-over that might be occurring of the excess thermospermine being produced. Currently we are pursuing the spatial localization of the *ACL5* transcript in poplar plants through *in situ* hybridization, and by taking advantage of the generated transgenic lines we hope to understand the role of thermospermine in the vascular tissues formation in this woody species.
